# Optimising amino acid absorption: essential to improve nitrogen balance and metabolic control in phenylketonuria

**DOI:** 10.1017/S0954422418000173

**Published:** 2018-10-04

**Authors:** Anita MacDonald, Rani H. Singh, Júlio César Rocha, Francjan J. van Spronsen

**Affiliations:** 1 Dietetic Department, Birmingham Children’s Hospital, Steelhouse Lane, Birmingham B74 4XN, UK; 2 Department of Human Genetics, Emory University School of Medicine, Nutrition Health Sciences Program, Emory University, Atlanta, GA, USA; 3 Medical Genetics Centre, Centro Hospitalar do Porto (CHP), Porto, Portugal; 4 Hereditary Metabolic Disease Reference Centre, Centro Hospitalar do Porto (CHP), Porto, Portugal; 5 Health Sciences Faculty, Universidade Fernando Pessoa, Porto, Portugal; 6 Centre for Health Technology and Services Research (CINTESIS), Porto, Portugal; 7 Division of Metabolic Diseases, Beatrix Children’s Hospital, University Medical Center Groningen, University of Groningen, Groningen, the Netherlands

**Keywords:** Phenylalanine, Phenylketonuria, Amino acids, Absorption, Amino acid mixtures, Protein synthesis, Nitrogen balance

## Abstract

It has been nearly 70 years since the discovery that strict adherence to a diet low in phenylalanine prevents severe neurological sequelae in patients with phenylalanine hydroxylase deficiency (phenylketonuria; PKU). Today, dietary treatment with restricted phenylalanine intake supplemented with non-phenylalanine amino acids to support growth and maintain a healthy body composition remains the mainstay of therapy. However, a better understanding is needed of the factors that influence N balance in the context of amino acid supplementation. The aim of the present paper is to summarise considerations for improving N balance in patients with PKU, with a focus on gaining greater understanding of amino acid absorption, disposition and utilisation. In addition, the impact of phenylalanine-free amino acids on 24 h blood phenylalanine/tyrosine circadian rhythm is evaluated. We compare the effects of administering intact protein *v.* free amino acid on protein metabolism and discuss the possibility of improving outcomes by administering amino acid mixtures so that their absorption profile mimics that of intact protein. Protein substitutes with the ability to delay absorption of phenylalanine and tyrosine, mimicking physiological absorption kinetics, are expected to improve the rate of assimilation into protein and minimise fluctuations in quantitative plasma amino acid levels. They may also help maintain normal glycaemia and satiety sensation. This is likely to play an important role in improving the management of patients with PKU.

## Introduction

Phenylalanine hydroxylase (PAH) deficiency^(^ ^1^ ^)^, also known as phenylketonuria (PKU), causes accumulation of neurotoxic levels of the substrate phenylalanine (Phe) and a relative deficit of the product tyrosine (Tyr)^(^ ^2^ ^)^. Unless treated early and continuously with a Phe-restricted and Tyr-supplemented diet^(^ ^3^ ^)^, children experience cognitive impairment^(^ ^4^ ^)^, while adolescents and adults may develop deficits in executive function, attention and social skills^(^ ^5^ ^–^ ^8^ ^)^. Dietary treatment with restricted Phe intake remains the mainstay of therapy for PKU, although a subset of patients usually with mild or moderate PKU may respond to treatment with pharmacological doses of the PAH cofactor tetrahydrobiopterin (BH_4_)^(^ ^3^ ^,^ ^9^ ^)^. The aim of the present paper is to review considerations for improving N balance in patients with PKU, with a focus on gaining a better understanding of amino acid absorption, disposition and utilisation.

## Dietary management of phenylketonuria

### Aims of dietary treatment

The primary aim of dietary treatment is to prevent adverse neurocognitive and psychological outcomes by restricting and titrating the intake of protein from natural foods to maintain blood Phe levels within the guideline-established range that prevents adverse outcomes while providing enough Phe to support protein synthesis and avoid catabolism^(^ ^3^ ^,^ ^9^ ^)^. There is general consensus on the importance of meticulous control of blood Phe levels within a narrow target range from birth to 12 years of age (120–360 μmol/l)^(^ ^3^ ^,^ ^9^ ^,^ ^10^ ^)^. For patients above the age of 12 years (exception pre-conception and pregnancy), the European guidelines consider 120–600 μmol/l to be safe throughout adolescence and adulthood^(^ ^3^ ^)^, while US guidelines advocate 120–360 μmol/l throughout life^(^ ^10^ ^)^. Protein substitutes have an important role in helping achieve optimal metabolic control.

The second aim of dietary management is to maintain a healthy nutritional status by providing sufficient non-Phe amino acids, energy and other nutrients to support physiological protein synthesis and counterbalance catabolism, without providing excess energy. N and essential amino acid requirements are met with a Phe-free amino acid mixture containing vitamins and other essential nutrients and the addition of special low-protein modified foods provides a necessary energy source^(^ ^11^ ^,^ ^12^ ^)^. The specialist dietitian and metabolic physician manage dietary treatment to maintain appropriate blood Phe levels in response to metabolic changes, while ensuring that other nutritional requirements are being satisfied to support normal growth and development in children and a healthy body composition in all patients^(^ ^13^ ^)^.

Notwithstanding the theoretical effectiveness of dietary treatment, its actual success depends on providing a diet that is acceptable and practical to the patient. Subjective factors that can hinder acceptance include poor palatability, disagreeable smell, or textures, lack of dietary variety and food neophobia^(^ ^14^ ^)^. Adherence with protein substitutes that must be consumed at least three times a day throughout life is a particular challenge. Socially acceptable, appropriate food choice is particularly important for improving adherence as patients move from childhood to adolescence, as they have more responsibility for managing their treatment and are exposed to new food choices and peer pressure outside the home^(^ ^15^ ^)^.

### Protein requirement

In PKU, when the protein requirement is met primarily with a Phe-free amino acid mixture, the protein equivalent amount of amino acids should be increased because of poor retention^(^ ^15^ ^)^. This higher requirement may have both a physiological cause, related to the oxidation of excess free amino acids due to their rapid, non-physiological absorption^(^ ^16^ ^–^ ^18^ ^)^, and perhaps a pathological cause, related to higher needs imposed by the condition itself, in the form of increased catabolism. Few studies have addressed this latter point. Van Rijn *et al.* ^(^ ^19^ ^)^ found no difference in protein metabolism between adults with PKU and matched healthy volunteers using an l-[1-^13^C]valine tracer method. In this study, six patients with PKU received amino acids equivalent to 0·96 g protein/kg per d (120 % of the WHO recommended daily allowance)^(^ ^20^ ^)^, while the six healthy volunteers received 0·8 g intact protein/kg per d (100 % of the recommended daily allowance). The 20 % increase in patients with PKU was administered to account for inefficient absorption/utilisation. The results suggest that, at least in adults with PKU, the increased requirement for amino acids results from reduced retention rather than an increase in metabolic requirement. In children, however, Turki *et al.* ^(^ ^21^ ^)^ used the indicator amino acid oxidation technique to study four patients with mild PKU ranging in age from 9 to 18 years, finding that their protein requirement was 1·85 g/kg per d, considerably higher than the recommended amount for healthy children^(^ ^21^ ^)^ and higher than the recommendation for children with PKU^(^ ^3^ ^,^ ^9^ ^,^ ^22^ ^,^ ^23^ ^)^. This may reflect methodological differences between older N balance studies that inform guidelines and the more sensitive isotope methods. Moreover, in a 6-week randomised, cross-over study conducted in twenty-five children aged 2–10 years (median 6 years), administering a higher dosage of protein substitute was associated with lower blood Phe levels^(^ ^24^ ^)^. Compared with a PKU diet containing the recommended protein requirement for children (1·2 g/kg per d), a similar diet with higher protein content (2·0 g/kg per d) resulted in median blood Phe levels that were 301 μmol/l lower when measured before breakfast (95 % CI 215, 386 μmol/l; *P*<0·001) and 337 μmol/l lower when measured in the evening (95 % CI 248, 431 μmol/l; *P*<0·001)^(^ ^24^ ^)^.

Either of these mechanisms (reduced retention or increased requirement) justifies the administration of increased amino acid intake or the use of an amino acid mixture formulated to achieve physiological absorption and avoid net protein catabolism, which causes the release of Phe and increases blood Phe concentrations. The current European guidelines suggest administering 140 % of the recommended daily requirement established for healthy individuals^(^ ^3^ ^)^, and the most recent US guidelines suggest administering 150 % until age 4 years and 120–140 % thereafter^(^ ^22^ ^)^.

## Digestion and absorption of intact protein

All amino acids contain at least one amine and one carboxyl group, and a specific side chain that determines the characteristics of the amino acid. In humans, eleven of the twenty standard amino acids are classified as either essential, because they cannot be synthesised (histidine, isoleucine, leucine (Leu), lysine, methionine, Phe, threonine, tryptophan and valine), or semi-essential, because they may not be synthesised sufficiently in growing children (cysteine and arginine); these must be obtained from the diet^(^ ^25^ ^)^. In addition to protein synthesis, amino acids such as glutamine, tryptophan and Tyr are precursors for neurotransmitters. Amino acids can also serve as an energy source^(^ ^26^ ^,^ ^27^ ^)^, although this is not their main role. The amino group is removed via transamination to α-ketoglutarate and then processed in the urea cycle. Amino acids are classified as either ‘glucogenic’, if their catabolism yields pyruvate or citric acid cycle intermediates that can generate glucose, or ‘ketogenic’ if their catabolism results in acetyl- or acetoacetyl-CoA.

Digestion of proteins starts in the stomach, where the low pH denatures them, removing their secondary structure and exposing them to cleavage by pepsin. Proteolytic enzymes of the exocrine pancreas (carboxypeptidase, chymotrypsin, elastase and trypsin) perform most of the digestion in the duodenum, producing short peptide fragments that are then processed into di- and tripeptides and free amino acids by aminopeptidase and dipeptidase on the apical membrane of enterocytes. Na-dependent amino acid transporters for acidic, basic, neutral and branched-chain amino acids utilise energy from the electrochemical Na gradient to transport their substrates across the apical membrane of the enterocyte.

Generally, several amino acids compete for binding to a shared transporter or exchanger, and different amino acids have different carrier affinities, depending on the mass of their side chain and the presence of an electrical charge. Affinity increases with mass and is also higher for neutral amino acids. The large neutral amino acids (LNAA) are transported by the LNAA transporter 1 (LAT1), also known as SLC5A7, for which Phe has very high affinity. Amino acids are absorbed also as di- and tripeptides through co-transport with H^+^ ions via peptide transporter 1 (PEPT 1; also known as solute carrier family 15 member 1 SLC15A1). With normal protein feeding, most amino acids enter the enterocytes from the intestinal lumen as peptides that are subsequently hydrolysed to free amino acids in the cytoplasm^(^ ^28^ ^)^.

Depending on the metabolic status of the enterocyte, absorbed amino acids may be utilised for energy, incorporated into proteins or released from the basolateral membrane into the hepatic portal circulation via Na-independent transporters. The N requirement of the intestinal mucosa is high, owing to rapid cell turnover, production of secretory proteins and a tendency to use amino acids as an energy source. The metabolic fate of an ingested N source depends also on the kinetics of amino acid absorption. Indications of this arise from comparing the so-called ‘fast’ and ‘slow’ proteins, named in this way because of their rates of digestion and amino acid absorption^(^ ^29^ ^,^ ^30^ ^)^. The digestion rate appears to be controlled by the rate of gastric emptying. Whey, a fast protein, remains soluble in the stomach and passes quickly to the intestine, whereas the slow protein casein coagulates and is retained longer^(^ ^31^ ^)^. Fast proteins result in less N assimilation^(^ ^29^ ^)^. This observation is confirmed by a study comparing the kinetics of dietary N after feeding either intact or hydrolysed casein protein labelled with ^15^N, which revealed reduced peripheral protein synthesis with hydrolysed casein (faster)^(^ ^32^ ^)^.

## Free amino acid supplementation

### Amino acid absorption

**Fig. 1:**
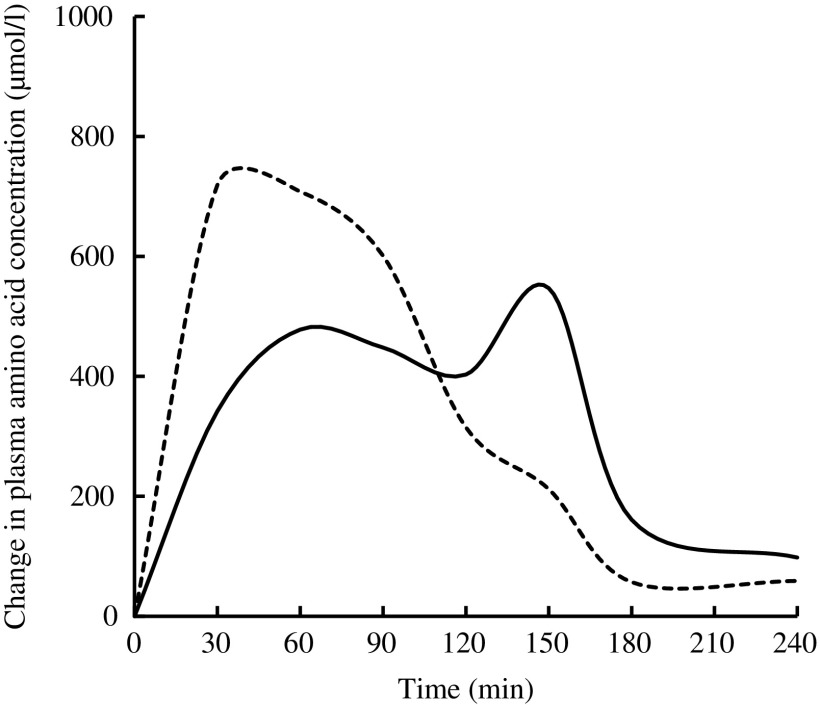
Mean change from baseline in total plasma amino acids after feeding whole protein (cottage cheese, ––) or an equivalent amount of a free amino acid mixture with identical amino acid composition (---) in fasting healthy volunteers (*n* 10). Adapted from Gropper & Acosta^(^ ^17^ ^)^.

Free amino acid mixtures are formulated to fulfil the specific requirements of patients with PKU. Administering free amino acids bypasses the digestive phase, and the absorption profile is different from that of intact proteins, in that plasma levels of total and essential amino acids are higher, peak faster and decrease more quickly (Fig. 1)^(^ ^17^ ^)^. Administering a free amino acid mixture with a prolonged release mimicking physiological absorption kinetics is expected to improve the rate of assimilation into protein and prevent fluctuations in plasma amino acid levels.

### Metabolic effects of amino acid absorption rate

Compared with physiological administration of free amino acid mixtures distributed through the day, bolus administration increases the amount of N excreted in urine^(^ ^18^ ^)^, when the rapid increase in blood amino acids exceeds the capacity of anabolic processes to incorporate them into nascent proteins. Dangin *et al.* ^(^ ^33^ ^)^ examined the effect of protein digestion rate on protein accretion using ^13^C-labelled Leu and various fast *v.* slow protein meals. They compared casein *v.* free amino acids mimicking casein, and whey protein bolus (fast protein) *v.* repeated dosing (simulated slow protein)^(^ ^33^ ^)^. Fast uptake was associated with a rapid increase in blood amino acids and more oxidation. Protein retention was greater with slow protein sources (casein *v*. free amino acids: 38 (se 13) *v.* –12 (se 11) µmol Leu/kg; *P*<0·01) and with repeated dosing *v*. whey bolus (87 (se 25) *v.* 6 (se 19) µmol Leu/kg; *P*<0·05). Jones *et al.* ^(^ ^34^ ^)^ compared continuous enteral nutrition with protein *v.* free amino acid mixtures in isonitrogenous, isoenergetic diets, and found that patients randomly assigned to receive the free amino acid mixture had significantly higher N loss^(^ ^34^ ^)^. However, a metabolic study comparing adult patients with PKU fed a protein-restricted, free amino acid mixture-supplemented diet with matched healthy subjects receiving a normal diet revealed no differences in whole-body protein metabolism at a protein intake of 0·8 g/kg per d^(^ ^19^ ^)^.

**Fig. 2:**
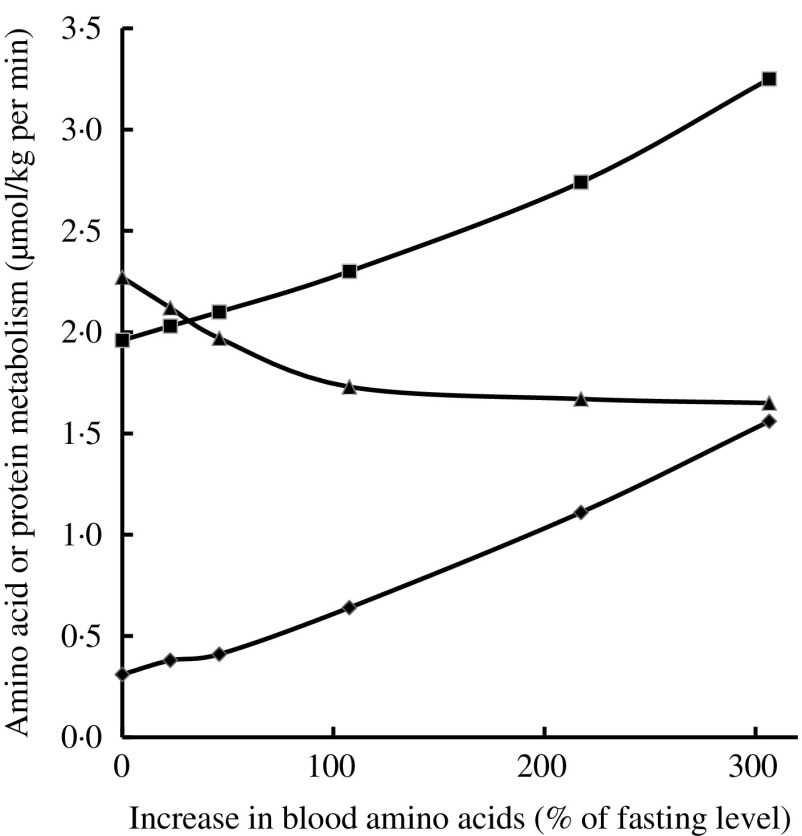
Effects of graded hyperaminoacidaemia on protein metabolism: a balanced amino acid solution was infused at 0·5, 1, 2, 4 and 6 mg/kg per min for 180 min in eight fasting healthy volunteers, and amino acid oxidation (–♦–), protein synthesis (–■–) and proteolysis (–▲–) were estimated with [1-^14^C]leucine infusion and indirect calorimetry. Redrawn from Giordano *et al.* ^(^ ^35^ ^)^.

Infusion of increasing amounts of a balanced mixture of free amino acids (from 0·5 up to 6 mg/kg per min) in fasted healthy young adults resulted in inhibition of protein catabolism, stimulation of protein synthesis and an increase in amino acid oxidation, depending on plasma amino acid levels attained (Fig. 2)^(^ ^35^ ^)^. Even small increases in amino acid concentrations compared with fasting concentrations inhibited protein catabolism and, in parallel, stimulated protein synthesis as well as amino acid oxidation. The decrease in protein catabolism was overruled by the increase in amino acid oxidation and most notably protein synthesis^(^ ^35^ ^)^.

Protein synthesis is stimulated both by amino acids and by insulin. When plasma amino acids levels are artificially maintained at post-absorptive levels (i.e. physiological hyperaminoacidaemia), administration of insulin further increases net protein synthesis and decreases protein catabolism, with no significant change in amino acid oxidation^(^ ^36^ ^)^.

Protein synthesis increases in the postprandial state because of anabolic signals from the insulin pathway and the increase in plasma amino acids, especially Leu, that work through the mammalian target of rapamycin (mTOR)^(^ ^37^ ^)^. Insulin and insulin-like growth factor 1 increase protein synthesis at the translation level via mTOR-mediated effects on components of the protein synthetic pathway, including the eukaryotic initiation factor 4E-binding protein 1 (4E-BP1) and the ribosomal protein S6 kinase (p70S6K), both key components of the translation initiation complex^(^ ^38^ ^)^. Thus, the anabolic signal from insulin, corresponding to abundant energy (glucose), is an important factor controlling protein metabolism^(^ ^39^ ^)^. This underscores the need for sufficient energy intake to promote anabolism and prevent an increase in N excretion when free amino acid mixtures are administered^(^ ^18^ ^,^ ^40^ ^,^ ^41^ ^)^. An optimal protein:energy ratio has been proposed as 3·0–4·5 g protein/100 kcal (418 kJ), corresponding to approximately 12–18 % of energy from protein^(^ ^42^ ^)^.

Amino acids themselves stimulate insulin secretion^(^ ^43^ ^)^. Branched-chain amino acids, especially Leu, have an insulinogenic effect when administered together with a glucose solution, compared with glucose solution alone^(^ ^44^ ^)^. Amino acids also stimulate the release of the incretins glucagon-like peptide-1 (GLP-1) and glucose-dependent insulinotropic polypeptide (GIP)^(^ ^45^ ^,^ ^46^ ^)^, which in turn increase the anabolic response by promoting insulin secretion^(^ ^47^ ^)^. The amino acid mixtures used in the dietary treatment of PKU contain a high level of Leu to promote anabolism. In healthy subjects, preloading with 9 g of either whey or soya protein, or a combination of the insulinogenic amino acids (isoleucine, Leu, lysine, threonine and valine) before a reference meal significantly reduced postprandial glycaemia. This correlated with increased incretin and insulin responses^(^ ^48^ ^)^. Compared with administration of free amino acid mixtures, physiological absorption of amino acids from intact protein represses ghrelin levels to a greater extent, providing a sense of satiety^(^ ^40^ ^,^ ^41^ ^)^.

Thus, in addition to favouring a positive N balance, more physiological absorption of amino acids in the dietary treatment of PKU may be expected to help maintain normal glycaemia and normal sensation of satiety. However, simulating normal absorption kinetics with existing free amino acid mixtures is problematic because it would complicate the treatment with more frequent dosing. When possible, administration of free amino acid mixtures should coincide with a postprandial state, in order to favour retention of dietary N.

## Disposition of key amino acids in phenylketonuria

### Role of the blood–brain barrier

The most severe pathological effects of PKU are in the brain. The blood–brain barrier (BBB) isolates the brain from fluctuations in blood levels of hormones, nutrients and metabolites, and provides the stable chemical environment required for delicate neural processes. The BBB is the result of tight junctions between the endothelial cells of brain capillaries and a multitude of transporters that regulate the passage of specific molecules. The concentration of essential amino acids inside the brain is approximately ten times lower than in the general circulation and is determined by the controlled transport of LNAA across the BBB^(^ ^49^ ^)^. This is accomplished through LAT1 (SLC7A5/SLC3A2 heterodimer), which has high affinity for the LNAA precursors important for neurotransmitter and protein synthesis^(^ ^50^ ^,^ ^51^ ^)^. LAT1 is a member of transporter system L, ubiquitous bidirectional Na-independent exchangers of branched and aromatic neutral amino acids^(^ ^52^ ^)^. In BBB endothelial cells, LAT1 is expressed on both the luminal (blood) and abluminal (brain) membranes and facilitates the transendothelial transport of LNAA (uptake substrates) in exchange for non-essential amino acid substrates (antiporter)^(^ ^53^ ^)^. Cells inside the brain have a three-fold higher affinity for essential amino acids compared with the BBB endothelia^(^ ^54^ ^)^, making the BBB the limiting transfer step. This system is nearly saturated at physiological blood amino acid concentrations, and is subject to substrate competition depending on LNAA abundance and affinity. Phe is the amino acid with the highest affinity for LAT1 and the high Phe level in PKU essentially blocks the uptake of other LNAA, reducing the synthesis of proteins and neurotransmitters in the brain^(^ ^55^ ^,^ ^56^ ^)^. The importance of the BBB and amino acid disposition as a mechanism in the pathophysiology of PKU is highlighted by the finding that a minority of patients with pathologically high blood Phe concentrations have lower than expected brain Phe levels and normal intelligence quotient (IQ) without treatment^(^ ^57^ ^–^ ^59^ ^)^; however, the mechanism for this rare and irregular outcome remains unidentified^(^ ^60^ ^)^.

### Large neutral amino acids

One approach to treating PKU involves administering higher than normal amounts of non-Phe LNAA in order to overcome this competition and allow other essential LNAA to enter the brain^(^ ^61^ ^)^. Decreasing Phe entrance into the brain by another LNAA would be accomplished most effectively with Leu (and isoleucine)^(^ ^62^ ^)^, but this does not address the entire pathogenesis of PKU brain dysfunction, and it might also result in increased brain Leu or isoleucine concentrations, which are both associated with inborn errors of metabolism with brain pathophysiology. Thus, the composition of LNAA in amino acid mixtures must be carefully balanced according to amino acid transporter affinities and importance for brain function. In particular, Tyr and tryptophan should be included at high levels to promote normal brain monoaminergic neurotransmitter concentrations^(^ ^62^ ^)^. Three mechanisms driving the improvement observed with LNAA administration have been confirmed in a murine PKU model: LNAA supplementation reduced brain Phe levels, increased brain levels of non-Phe LNAA and increased neurotransmitter levels^(^ ^63^ ^)^. However, dopamine levels were not normalised in this study, once again indicating the necessity to carefully balance the composition of the LNAA administered.

### Tyrosine

The product of PAH is Tyr, which becomes a conditionally essential amino acid in patients with PKU^(^ ^64^ ^)^. Tyr is essential for protein and dopamine synthesis in the brain. Patients with uncontrolled PKU have reduced levels of dopamine because Tyr is not produced from Phe and high blood Phe levels prevent exogenous Tyr from crossing the BBB. Tyr supplementation may cause blood Tyr levels to fluctuate greatly during the day in treated patients with PKU^(^ ^65^ ^–^ ^69^ ^)^. CV for blood Tyr concentrations are about ten-fold higher than those of Phe^(^ ^69^ ^)^. This may be due to the small pool of Tyr in patients with PKU and the relatively high level of Tyr in the free amino acid mixtures administered in the PKU diet^(^ ^69^ ^)^. It should be noted that, although the amino acid mixtures contain high levels of Tyr, it is not highly soluble, and solutions containing Tyr must be mixed well before administration. A slower release of free amino acids allowing a more physiological absorption may also reduce the amplitude of blood Tyr peaks and overall degree of fluctuation.

### Fluctuations in blood phenylalanine levels over 24 h

In healthy subjects, the blood concentrations of individual amino acids undergo circadian variation, with peak levels occurring in the evening^(^ ^70^ ^)^. This rhythm is stable and not influenced significantly by diet^(^ ^71^ ^)^. Much of the ingested Phe is converted to Tyr in the liver, stabilising blood Phe levels, and maintaining the amplitude of variation generally < 50 %^(^ ^72^ ^)^. Greater circadian fluctuations in blood Phe levels have been reported in patients with PKU^(^ ^65^ ^,^ ^73^ ^,^ ^74^ ^)^. These fluctuations may be more apparent with PAH genotypes that have low/no residual enzyme activity^(^ ^75^ ^)^. In addition, it is suggested that BH_4_ in pharmacological doses reduces Phe fluctuations in BH_4_-responsive patients; however, responsive patients have residual PAH activity with less severe PKU, and generally have lower and less variable blood Phe^(^ ^76^ ^,^ ^77^ ^)^.

The normal pattern of circadian fluctuations in blood Phe is inverted in patients with PKU, with the highest levels occurring in the morning instead because of catabolism triggered by overnight fasting^(^ ^65^ ^,^ ^74^ ^)^. Diurnal fluctuations may also result from inappropriate distribution of free amino acid mixtures during the day, with even distribution throughout the day resulting in more stable Phe levels^(^ ^73^ ^,^ ^78^ ^)^; however, administration during waking hours only is not sufficient to prevent nocturnal catabolism. Fluctuations in blood Phe from day to day, or over longer time-frames may result from inconsistent adherence to dietary treatment, failure to adjust treatment to changes in growth rate and catabolism associated with illness.

Although it has been suggested that Phe fluctuations may be associated with poorer outcomes, the mechanism by which fluctuations may affect outcomes is not known^(^ ^72^ ^)^. Fluctuations in Phe levels are implicated as a factor influencing IQ and cognitive performance in PKU^(^ ^6^ ^,^ ^79^ ^–^ ^81^ ^)^. In adults, stability of Phe levels may be as important as absolute Phe levels for cognitive outcomes^(^ ^6^ ^,^ ^81^ ^)^. International guidelines do not specifically address Phe fluctuations; however, consistently following management plans, attention to dietary intake during intercurrent illness and measuring Phe levels at the recommended intervals may reduce the frequency and magnitude of Phe fluctuations.

## Special considerations in phenylketonuria

### Improving compliance to amino acid feeding

Adherence with diet is fundamental to successful treatment of PKU, and non-adherence represents a major cause of blood Phe levels outside of recommended ranges^(^ ^73^ ^)^. Poor adherence can also have negative effects on overall health status if there is low and inconsistent intake of a macro- and micronutrient-supplemented amino acid mixture.

PKU represents a substantial burden^(^ ^82^ ^)^. The need to severely reduce the intake of protein-rich foods, access special low-protein modified foods, prepare special meals, and monitor and calculate the dietary protein/Phe intake can be particularly onerous for both carers and patients^(^ ^15^ ^,^ ^83^ ^–^ ^85^ ^)^. This is further compounded by the need to administer multiple daily doses of free amino acid mixtures that some patients find distasteful. Despite efforts to improve palatability, the development of an amino acid mixture with organoleptic properties acceptable to all patients remains a challenge. This is particularly important among older patients who have low adherence rates^(^ ^15^ ^)^, and may have stopped diet treatment because of difficulties in accepting and tolerating amino acid supplements.

### Phenylalanine tolerance

Depending on the amount of residual PAH activity associated with a patient’s genotype, there is considerable variation in the amount of natural protein (Phe) that patients can tolerate while maintaining blood Phe levels in the recommended range. Increasing Phe tolerance may improve adherence to treatment and quality of life by allowing the intake of more natural foods. A subgroup of patients with PKU, comprising mainly those with mild/moderate disorder, is responsive to pharmacological treatment with BH_4_. This can minimise dietary restrictions by allowing higher intake of natural protein^(^ ^86^ ^,^ ^87^ ^)^; however, there is a risk of micronutrient deficiencies due to the decrease in the intake of vitamin and mineral-supplemented Phe-free l-amino acid mixtures^(^ ^88^ ^)^. Careful monitoring is still required to ensure that the diet is nutritionally adequate, and long-term longitudinal studies are needed to understand the real impact of BH_4_ on general nutritional status.

## Towards a physiological amino acid absorption

Two different approaches have been hypothesised to deliver free amino acid mixtures with physiological absorption kinetics: existing free amino acid mixtures could be administered throughout the day, or methods could be developed to prolong the gut transit time and/or absorption kinetics of the amino acid mixture to simulate the physiological absorption of intact protein.

The first approach involves giving a more evenly distributed amino acid mixture over 24 h, including a nocturnal dose^(^ ^78^ ^)^. However, this will create problems with adherence by complicating treatment because of the increased number of doses to plan and remember, going against the unmet needs and desires expressed by patients and carers to improve quality of life by simplifying therapy and normalising the social context of treatment^(^ ^89^ ^–^ ^91^ ^)^.

The second approach requires the development of a sustained-release formulation that provides a physiological absorption profile similar to that of intact natural proteins, or a controlled-release system that maintains blood amino acid levels above a certain threshold for an extended period. This would not only improve the balance between anabolism and catabolism induced by amino acids, but ideally might also simplify therapy by reducing the number of amino acid mixture administrations. However, such technology must not have deleterious effects on overall digestion. Moreover, regardless of the strategy used to modify amino acid absorption, care must be taken not to disrupt the intricate amino acid signalling mechanisms involved in digestive secretion, motility and the gut–brain axis.

## Discussion

Despite recent improvements, more than half of patients with PKU report that managing their disorder is ‘difficult’. Moreover, although it has been recommended in the US guidelines since 2000, a recent study showed that one in four patients < 18 years of age do not maintain Phe levels < 360 μmol/l, and non-controlled adults constitute almost two-thirds of the surveyed PKU population^(^ ^91^ ^)^. Surveyed patients identified neurocognitive issues with attention, memory, executive functions, depression, anxiety and mood disturbances among the most important symptoms to improve^(^ ^91^ ^)^. Whether physiological absorption of amino acids has any effects on Phe-level control remains to be demonstrated; however, even a small improvement in Phe tolerance would be especially relevant for patients with very high untreated blood Phe levels. The aim of the present review was to discuss how an improved N balance in patients with PKU might theoretically make an impact on patients, and how such a goal may be reached by modifying the absorption of the free amino acids that patients with PKU utilise every day. However, this issue is also relevant to other conditions that require a protein-restricted diet supplemented with a specific amino acid mixture, such as tyrosinaemia, maple syrup urine disease and perhaps homocystinuria.

Amino acid absorption profiles represent an area of disease control that has received little attention. We believe that prolonging the absorption of free amino acids may help to maintain a positive N balance and reduce catabolic episodes during the 24 h cycle, supporting optimal growth in children and the maintenance of muscle mass in adults. Better utilisation of amino acids entering the blood gradually might sustain more efficient anabolism and at the same time reduce the amount of amino acids that are oxidised because of the sudden excess of these nutrients after the consumption of a protein substitute dose. This hypothesis is supported by studies that suggest that the accumulation of lean mass is associated with the amount of intact protein in the PKU diet^(^ ^92^ ^,^ ^93^ ^)^. However, in the study by Jani *et al.* ^(^ ^93^ ^)^, improvements in lean body mass and the ratio of lean:fat body mass seen with a higher intake of intact protein were accompanied by high mean blood Phe values of 870μmol/l among adults and 613μmol/l among children. Therefore, simply increasing the intake of typical intact protein is not a viable solution. An ideal protein source for PKU would be a ‘slow’ protein with no or very low levels of Phe, high levels of Tyr and perhaps other LNAA, and a normal composition of the other amino acids. Such a protein would not require supplementation with free amino acids, other than Phe. A natural protein with these characteristics has not been identified and producing it through recombinant methods would not be economically feasible; in either case, it would need to be purified. Glycomacropeptide (GMP), a sixty-four-amino acid peptide released from casein during cheese production, has some of the characteristics sought. However, commercial GMP does contain a small amount of Phe. Also, because pure GMP lacks the essential amino acids histidine and tryptophan, the semi-essential amino acids arginine, and cysteine, and the conditionally essential amino acid Tyr, they must be added as free amino acids to GMP protein substitutes. GMP is a small acid-soluble peptide isolated from the whey fraction, and is thus a ‘fast’ protein. Nonetheless, results of a study in eleven patients revealed lower blood urea N and higher postprandial blood amino acid levels compared with the standard PKU diet supplemented with a free amino acid mixture^(^ ^94^ ^)^, suggesting that a strategy providing physiological absorption of all non-Phe amino acids is worth pursuing.

Modifying the disposition and distribution of amino acids to resemble what occurs after ingestion of intact protein may promote a balanced transport of LNAA across the BBB for extended periods, thereby contrasting the preferential influx of Phe into the brain because of its high affinity for the LAT1 transporter. As a consequence, brain essential amino acid concentrations may be more balanced, with an effect on neurotransmitter synthesis and in turn plausible positive consequences on neurocognitive outcomes.

In addition to existing strategies to relieve the burden of PKU, including supplements with improved palatability, GMP-based products, LNAA supplementation and BH_4_ therapy for those who respond, the availability of an amino acid mixture with absorption-prolonging features warrants further investigation and validation of clinical benefits in PKU patients.
